# Multiphysics and Multiscale Analysis for Chemotherapeutic Drug

**DOI:** 10.1155/2015/493985

**Published:** 2015-09-28

**Authors:** Linan Zhang, Sung Youb Kim, Dongchoul Kim

**Affiliations:** ^1^Department of Mechanical Engineering, Hangzhou Dianzi University, Hangzhou 310018, China; ^2^School of Mechanical and Advanced Materials Engineering, Ulsan National Institute of Science and Technology, Ulsan 689-798, Republic of Korea; ^3^Department of Mechanical Engineering, Sogang University, 1 Shinsoo-dong, Mapo-go, Seoul, Republic of Korea

## Abstract

This paper presents a three-dimensional dynamic model for the chemotherapy design based on a multiphysics and multiscale approach. The model incorporates cancer cells, matrix degrading enzymes (MDEs) secreted by cancer cells, degrading extracellular matrix (ECM), and chemotherapeutic drug. Multiple mechanisms related to each component possible in chemotherapy are systematically integrated for high reliability of computational analysis of chemotherapy. Moreover, the fidelity of the estimated efficacy of chemotherapy is enhanced by atomic information associated with the diffusion characteristics of chemotherapeutic drug, which is obtained from atomic simulations. With the developed model, the invasion process of cancer cells in chemotherapy treatment is quantitatively investigated. The performed simulations suggest a substantial potential of the presented model for a reliable design technology of chemotherapy treatment.

## 1. Introduction

Palliative chemotherapy has demonstrated improvements in survival and clinical trials for cancer patients [1]. The effectiveness of chemotherapy depends upon various factors including the type of cancer and location of cancer cells in the body [2]. The estimation of effectiveness is moreover ambiguous since chemotherapeutic drug that destroys abnormal or cancer cells also damages host cells. Because of the low efficacy of current treatments for metastatic disease, there is considerable interest in developing new systems to test chemotherapeutic drugs more quickly and accurately [3].

In the late 1980s, the National Cancer Institute (NCI) developed an in vitro drug discovery tool to test new therapeutics using 60 different human cancer cell lines [4]. The ability to test new anticancer therapeutics offers a great advantage in the development of therapies to treat cancer cells. Some reports indicated that many cancer chemotherapeutic agents are not usually selectively delivered to tumor tissues and normal cells are also destroyed by the drug [2, 5]. When administered systemically, these agents are distributed to both normal and tumor tissues by normal diffusion through capillaries [6]. An in vitro experiment has shown that the transport of small drug molecules through surrounding normal tissue is generally considered to be relatively faster than in cancer cells which are densely packed in islet [7, 8]. This finding demonstrated the reason why normal cells are destroyed faster than cancer cells by the drug. Thus, drug treatment strategy is very crucial in cancer chemotherapy and contemporary preclinical models have employed it in cancer drug development both in vitro and in vivo [5, 9]. Mathematical models of chemotherapeutic drug have been suggested to design treatment strategies that effectively destroy tumor cells while limiting toxicity on normal cells [10–14]. One of the mathematical models aimed to minimize the total amount of drug used such that the tumor cell population at the end of the treatment period reaches a specified value [11]. But it is difficult to correctly formulate and use the constraint expressing toxicity due to the cumulative drug. Recently, simulations have been performed with some proposed models [15–23]. They introduced that cancer cells had an ability to degrade and migrate into surrounding extracellular matrix (ECM). Each component involved in invasive process of tumor cells was considered as individual identity, and the model incorporated only cell-cell interaction excluding the effects of cell-matrix interaction [15]. The other mathematical models were developed to study the interaction of cancer cells with surrounding matrix [16]. But this model did not directly incorporate the interaction between cancer cells and ECM. They assumed that invasive process is triggered by cancer cells releasing matrix degrading enzymes (MDEs) which modify the ECM by degrading it. They made the effort on studying the interaction between MDEs and the degraded ECM. Byrne et al. presented a simple one-dimensional model of trophoblasts invading the uterine tissue to study placental development [17]. They explained the dominant migratory mechanism without any morphological analysis in the process of invasion. Harley et al. discussed the existence of traveling wave solutions of a haptotaxis model for malignant tumor invasion [18]. This work did not consider any energetic aspects such as surface or interface energy, which limits the accuracy of the solution. Andasari et al. studied the process of cancer-cells invasion based on mathematical analysis and computational simulation [19]. This work offered us a perspective on the ability of cancer cells to break out of tissue compartments and invade local tissue. Their simulation results were obtained by one- and two-dimensional models that limited the accurate and comprehensive understanding of the invasion process of cancer cells. In our work, a three-dimensional model incorporates multiple kinetics and energetics is developed to overcome the aforementioned limits on understanding the mechanism and morphological strategy of cancer-cells invasion. Moreover, a multiscale approach is employed, which increases the accuracy of simulation results by providing material properties with atomic simulations.

Here, we propose a three-dimensional dynamic model for the chemotherapy design. The model incorporates multiple components to study interactions between cancer cells, MDEs, degrading ECM, and chemotherapeutic drug. Multiple mechanisms possible in chemotherapy are systematically integrated for the high possibility of adequate predictions. Migration of cancer cells driven by haptotaxis, MDEs secreted by cancer cells, degradation of extracellular matrix, and diffusion of chemotherapeutic drug are analyzed simultaneously by considering multiple mechanisms in cancer-cells invasion. The velocity, number, and morphological changes of cancer cells are affected by diffusion of chemotherapeutic drug. Meanwhile, the degradation of the ECM is influenced by the diffusion of chemotherapeutic drug and MDEs which are secreted by cancer cells. Furthermore, a multiscale approach that links atomic-scale information to macroscopic properties of drugs is adopted. Molecular dynamic (MD) simulations have been carried out to examine the diffusion of chemotherapeutic drug and enhance the fidelity of the developed model. The evolving morphologies of the microstructures related to multicomponents, multiphysics, and multiscales cause computational challenges. These are addressed by employing a diffuse interface model and the reliability and effectiveness of the model are demonstrated. A series of simulations are performed to systematically investigate the effects of chemotherapeutic drugs on cancer cells and the ECM.

## 2. Materials and Methods

### 2.1. Multicomponents Model

**Figure 1:**
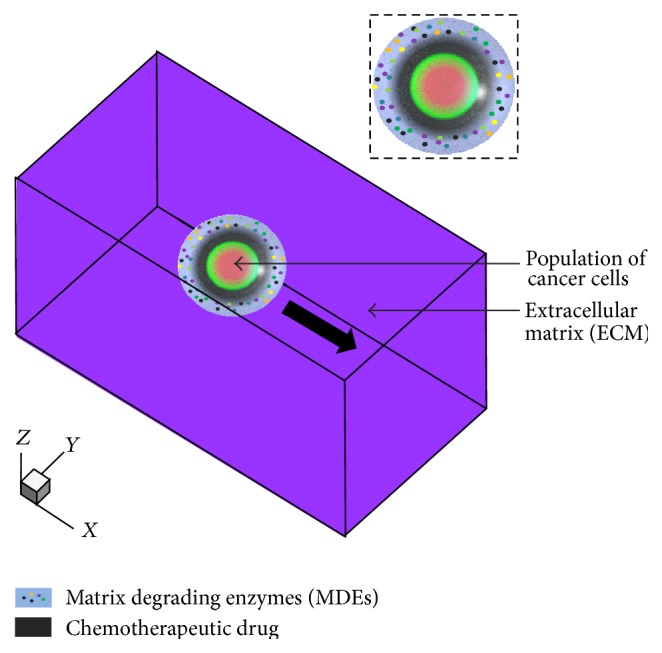
Schematic drawing represents the process of cancer-cells invasion with chemotherapeutic drug.

A diffuse interface model has been employed to efficiently present the morphological evolution of the microstructure system, which incorporates multiple kinetics and energetics in the multicomponent system, and demonstrated its reliability and effectiveness [24–28]. We develop the diffuse interface model that incorporates a series of multicomponents, which play decisive roles in the invasion process and chemotherapy. The model incorporates multiple kinetics that are the interdiffusion of chemotherapeutic drug and cancer-cell migration induced by haptoattractant. Multiple energetics are also incorporated, which are the interface energy among the population of cancer cells, the interface energy of the ECM, and the interface energy between cancer cells and the ECM. The phase field equations with multicomponents are driven by the reduction in the total free energy of an inhomogeneous system. The multicomponents of cancer-cell invasion with chemotherapeutic drug are displayed in the schematic drawing of Figure 1. We define a field variable *c*_1_ by the volume fraction of cancer cells, which describes the structural domain of cancer cells in the simulations. *c*_1_ = 1.0 corresponds to the pure cancer cells and *c*_1_ = 0.0 to other materials such as the ECM. Define a field variable *c*_2_ by a volume fraction of the ECM. *c*_2_ = 1.0 corresponds to the pure ECM and *c*_2_ = 0.0 to other materials such as cancer cells, which is dimensionless. The observed morphology of the ECM from experiments will correspond to the space *c*_2_ = 1.0 in the simulation results. *m* is defined by the concentration of the MDEs and *θ* by concentration of chemotherapeutic drug. In our work, we use four variables, *c*_1_(*x*, *y*, *z*, *t*), *c*_2_(*x*, *y*, *z*, *t*), *m*(*x*, *y*, *z*, *t*), and *θ*(*x*, *y*, *z*, *t*), which are time dependent and spatially continuous functions to describe cancer cells, MDEs, ECM, and chemotherapeutic drug. As an initial condition, we assume that *θ* corresponds to initial concentration of chemotherapeutic drug performed in the model. Various initial concentrations of chemotherapeutic drug 0.0; 5.0 × 10^−4^; 1.0 × 10^−3^; 5.0 × 10^−3^ are tested to provide analytical information of the capability of the drug in cancer treatment. The population of cancer cells is driven by haptotaxis in the model. Haptotaxis is the directional migration of the cells due to increasing haptoattractant gradients mediated by specific material. Fibronectin is indicated to be a chemoattractant or haptoattractant for certain tumor cells [29, 30]. The maximum concentration of fibronectin is assigned on the right region of the ECM. The linear haptoattractant gradient field is introduced only along the *x* direction to ignore the effect of the surrounding boundary on the cancer-cells invasion. Driving mechanisms for the evolution of each component as well as the interactions of them are incorporated in the following sections.

### 2.2. Multiphysics Model

A three-dimensional dynamic model based on multiphysics technology to study the process of cancer-cells invasion with chemotherapy is expounded here. The morphological evolution of cancer cells and the ECM are represented by the free energy composed of the respective field variables, *c*_1_ and *c*_2_. From the general diffuse interface approach, the total free energy of the microstructure system is followed by Cahn-Hilliard model [31] and then given by

(1) G=∫Vfc1,c2+h11∇c12+h22∇c22+h12∇c1∇c2dV.

In order to describe each component, the term *f*(*c*_1_, *c*_2_) could be any function with double wells. In numerical simulations, term *f*(*c*_1_, *c*_2_) with three components is derived as [32]

(2) fc1,c2=f0c121−c12+c221−c22+c321−c32+a1·c124151−c11+c1−c3−c22+c19c12−5+a2·c224151−c21+c2−c3−c12+c29c22−5+a3·c324151−c31+c3−c1−c22+c39c32−5,

where *f*_0_ is a positive constant, a domain without cancer cells, and ECM is represented by *c*_3_, which is equal to 1 − *c*_1_ − *c*_2_. We assume *a*_1_ = *a*_2_ = *a*_3_ = 1 for simple calculations. The remaining terms illustrate the interface energy among components. *h*_11_, *h*_12_, and *h*_22_ are gradient energy coefficients. The net fluxes of *c*_1_ and *c*_2_ are given by **J**_1_ = −*M*_1_∇*μ*_1_^0^ + *M*_1_ *β*∇*ϕ*, **J**_2_ = −*M*_2_∇*μ*_2_^0^, and the details of the derivation of these expressions are represented in the following paragraphs.

Chemical potentials *μ*_1_^0^ and *μ*_2_^0^ are related to the free energy of the system and defined by *μ*_*i*_^0^ = *δG*/*δc*_*i*_. Driving forces for the evolution of each component are attributed to the chemical potential by **F**_*d*1_ = −∇*μ*_1_^0^ and **F**_*d*2_ = −∇*μ*_2_^0^. We represent fluxes of *c*_1_ and *c*_2_ as **J**_*d*1_ = −*M*_1_∇*μ*_1_^0^ and **J**_*d*2_ = −*M*_2_∇*μ*_2_^0^. Here *M*_1_ and *M*_2_ are mobilities of cancer cells and the ECM. The mobility of cancer cells is described by *M*_1_(*c*_1_) = *M*_0_[{∫*c*_1_^2^(1 − *c*_1_)^2^ *dc*_1_/∫_0_^1^ *c*_1_^2^(1 − *c*_1_)^2^ *dc*_1_}(1 − *c*_1_)] and will vanish outside the interfacial region of *c*_1_ [24, 28, 33]. The mobility has a strong dependence on the local structure and the variable *c*_1_. The function of mobility instead of a constant mobility is employed and defined considering the excellent numerical stability [33], at the same time, following the model for the taxis phenomenon, which is developed by Keller and Segel [34]. The flux of *c*_1_ is additionally induced by external stimuli and given by **J**_*c*_ = *χc*_1_∇*ϕ*, where *χ* is haptotaxis sensitivity. *ϕ* is haptoattractant density, which represents external stimuli to provide the driving force ∇*ϕ* for cancer-cells directional migration. We employed a function with a linear gradient as *ϕ* in our simulations. *M*_1_ *β* corresponds to the haptotaxis sensitivity since *χ* is proportional to the mobility of cancer cells [35], where *β* is the sensitivity constant. The additional flux of cancer cell induced by haptotaxis can be rewritten as **J**_*c*_ = *M*_1_ *β*∇*ϕ*. Thus, the final net flux of *c*_1_ and *c*_2_ can be expressed as **J**_1_ = **J**_*d*1_ + **J**_*c*_ and **J**_2_ = **J**_*d*2_:

(3) J1=−M1∇μ10+M1β∇ϕJ2=−M2∇μ20.

The evolution of each field variable *c*_1_ and *c*_2_ is governed by Cahn-Hilliard nonlinear diffusion equation and combined with mass conservation relation ∂*c*_1_/∂*t* = −∇·**J**_1_, ∂*c*_2_/∂*t* = −∇·**J**_2_. The expressions of *c*_1_ and *c*_2_ are obtained as follows:

(4) ∂c1∂t=−∇·−M1∇μ10+M1β∇ϕ∂c2∂t=−∇·−M2∇μ20.

MDEs secreted by cancer cells and the chemotherapeutic drug are modeled as diffusing throughout the tissue and undergoing some form of decay. The kinetic process of MDEs and chemotherapeutic drug in the invasion process are described as follows:

(5) ∂m∂t=∇·M3∇m+εc1−λm

(6) ∂θ∂t=Mdrug∇2θ−ηθ,

where *M*_3_ is the mobility of MDEs and *ε* represents the released rate of MDEs by cancer cells. *λ* is the natural degradation rate of the enzymes. *M*_drug_ is the diffusion coefficient of the drug, which is obtained from the atomic calculations. The drug diffuses throughout cancer cells and the tissue and undergoes some form of decay related to *η*.

### 2.3. Multiscale Model

The derived governing equations are based on the continuum framework, which allows us to acquire a systematic picture of the invasive process of cancer cells and practically design the effective chemotherapy. To achieve the atomistic accuracy and broaden the applicability of the model, we employ a multiscale approach that bridges the gap between the atomic-scale information and the continuum analysis by transferring the calculated characteristics of the system with atomic simulations to the continuum calculation. Atomic simulations could provide information on calculating static and dynamic properties such as diffusion coefficient [36]. The diffusion of chemotherapeutic drug is investigated with atomic-scale calculations to enhance the possibility of expansion to various chemotherapeutic drugs.

The diffusion coefficient of chemotherapeutic drug in a polymer has been calculated from MD simulations. A cellular biological medium consists of extracellular space and extracellular polymer fibers. The amorphous polymer (polydimethylsiloxane, PDMS) is thus taken as a diffusion medium, which represents the ECM in our atomic simulations, and doxorubicin (C27H29NO11) that is chemotherapeutic drug and regarded to diffuse in PDMS. Doxorubicin is one of the most frequently used anticancer drugs in cancer treatment [37]. The diffusion coefficient is unique parameter for each specific drug and denotes the capacity of the drug diffusing in a specified medium, which is primary depending on its own chemical structure and affected by the external conditions such as the medium and temperature. Thus, the required information for calculation is the chemical structure of doxorubicin, PDMS, and temperature. Our atomic simulation is achieved with the structure of systems in a constant volume and constant density of the molecules at room temperature. Here we use a system consisting of five chemotherapeutic drug (doxorubicin) molecules and ten polydimethylsiloxane (PDMS) molecules. All the molecules are packed together and rotated. The three-dimensional drug-polymer structures are constructed by using geometry optimization method. Periodic boundary conditions are imposed in the model. Simulations are running at the temperature of 298 K with a constant volume. Then the whole structure is allowed to optimize the energy and equilibrated at the certain temperature. Purpose of the energy minimization is to ensure that dynamic simulation starts with a relatively stable structure.

Dynamic simulation begins with the optimized structure and the simulation time is selected to be 15 ps. Li et al. pointed out that value of diffusion coefficient decreased as the simulation time increased and the calculated results were consistent with the experimental value when the simulation time was between 10 ps and 20 ps [38]. In a short simulation time, the mean-square displacements show a nonlinear function with respect to the time and this tends to overestimate the diffusion coefficient [39]. With longer simulation time, the diffusion coefficient will not be changed considerably. Thus, the dynamic simulation time is selected to be 15 ps. In MD simulations, the diffusion coefficient of the drug, which is obtained from the calculated trajectory of it, can be defined as [38]

(7) $$\begin{array}{c} M_{drug}=\frac{1}{6N}\underset{i\to \infty }{\lim}\frac{d}{dt}\overset{N}{\underset{i=1}{\sum }}\langle \;{\left[\;r_{i}\left(\;t\;\right)-r_{0}\left(\;t\;\right)\;\right]}^{2}\;\rangle , \end{array}$$

where *M*_drug_ is the diffusion coefficient of the drug. *N* is the total number of drug molecules. The angular brackets represent mean-square displacement (MSD) of the drug. Diffuse behavior can be recorded in a trajectory file for each time step of dynamic simulation. MSD obtained from the trajectory of the drug molecules, which is a time dependent function, represents an average of squared distances summed up over all possible positions of the origin. *M*_drug_ is proportional to the slope of MSD.

The diffusion coefficient of a material diffusing in a specified medium can be affected by external conditions such as concentration of the medium and temperature. We consider constant external conditions and corresponding constant diffusion parameter, which is obtained by MD simulations before the macroscopic migration simulations, to obtain the sufficient efficiency of simulations for the practical analysis. Meanwhile, thirty MD simulations with the same initial conditions have been performed to achieve the accurate average value of diffusion coefficient. Once diffusion coefficient is calculated from MD simulations, the calculated diffusion coefficient is employed to the phase field model to investigate dynamic evolution of chemotherapeutic drug. In phase field model, *θ* denotes the volume fraction of chemotherapeutic drug. As an initial condition, *θ* corresponds to initial concentration of chemotherapeutic drug considered in the model. The diffusion coefficient of chemotherapeutic drug is space and time dependent function and defined by the field variables of *c*_1_ and *c*_2_, *M*_drug_(*θ*) = *M*_drug−*i*_∑_*i*=1_^*K*^(1/*K*)[{∫*c*_*i*_^2^(1 − *c*_*i*_)^2^ *dc*_*i*_/∫_0_^1^ *c*_*i*_^2^(1 − *c*_*i*_)^2^ *dc*_*i*_}(1 − *c*_*i*_)] [24, 28, 33]. The diffusion coefficient of chemotherapeutic drug in the ECM is obtained from MD simulations. There is no viable result to investigate the diffusion coefficient of chemotherapeutic drug in cancer cells. Some experimental observations indicated that drug molecules diffuse with the higher diffusivity in the ECM than in cancer cells [7, 8]. Suppose that the diffusion coefficient of chemotherapeutic drug in cancer cells is approximately 50% of that in the ECM. *M*_drug_ is a dimensionless number normalized by the calculated diffusion coefficient that is obtained from MD simulations.

From the experiment, it is introduced that the treatment of solid tumors is difficult to achieve the desirable results because the chemotherapeutic drug reaching to target cells is based on diffusion [6]. The diffusion coefficient of a particular anticancer drug in a specified medium provides information about measuring whether the drug would diffuse in a favorable state. In in vitro or in vivo systems, the measurement of delivery of chemotherapeutic drug at the precise site can be facilitated by the computational results. From a targeting perspective, providing the different diffusion coefficients of chemotherapeutic drug in different tissues is useful in improving clinical treatment on tumor cells.

### 2.4. Numerical Implementation

The process of cancer cell invasion consists of two steps; cancer cells produce MDEs and the ECM is degraded by MDEs. Meanwhile, it is well known that cancer chemotherapy is a systemic treatment. When chemotherapeutic drug is introduced, both cancer cells and the ECM are destroyed [2, 5, 6]. However, the ECM is degraded not only by the drug but also by MDEs that are secreted by cancer cells. Before the numerical discretization of the derived equations, we assume that *μ*_1_ = *μ*_1_^0^ − *βϕ* = *δG*/*δc*_1_ − *βϕ* = (2*c*_1_ + *c*_2_ − 1)(2*c*_1_^2^ − 2*c*_1_ + 2*c*_1_ *c*_2_ − *c*_2_ + 2*c*_2_^2^) − *ch*_11_^2^∇^2^ *c*_1_ − (1/2)*ch*_12_^2^∇^2^ *c*_2_ − *αϕ*; here, *α* = *βϕ*_0_/2*f*_0_ represents the significance of the haptotaxis. Normalize the governing equations of each multicomponent with a characteristic length *L*_*c*_ and time *t*_*c*_ = *L*_*c*_^2^/*M*_0_ *f*_0_. The normalized equations of cancer cells and ECM are given by

(8) ∂c1∂t=∇·M1∇μ1−ηθc1

(9) ∂c2∂t=∇·M2∇μ2−γmc2−ηθc2,

where *μ*_1_ = (2*c*_1_ + *c*_2_ − 1)(2*c*_1_^2^ − 2*c*_1_ + 2*c*_1_ *c*_2_ − *c*_2_ + 2*c*_2_^2^) − *ch*_11_^2^∇^2^ *c*_1_ − (1/2)*ch*_12_^2^∇^2^ *c*_2_ − *αϕ* and *μ*_2_ = *μ*_2_^0^ = *δG*/*δc*_2_ = (*c*_1_ + 2*c*_2_ − 1)(2*c*_1_^2^ − *c*_1_ + 2*c*_1_ *c*_2_ − 2*c*_2_ + 2*c*_2_^2^) − *ch*_22_^2^∇^2^ *c*_2_ − (1/2)*ch*_12_^2^∇^2^ *c*_1_. *γ* is the degradation rate of the ECM by MDEs. The last terms of (8) and (9) denote the capacity of chemotherapeutic drug in destroying cancer cells and normal cells, respectively. The initial haptoattractant density *ϕ* and the mobility *M*_1_, *M*_2_, *M*_3_ are dimensionless numbers normalized by *ϕ*_0_ and *M*_0_. The values of the parameters used in the multiscale system are listed in Table 1. The significance of the interface energy between the components is described by the Cahn numbers, *ch*_*ij*_^2^ = *h*_*ij*_/*L*_*c*_^2^ *f*_0_. The choice of the magnitudes of the characteristic quantities depends on physical details to be resolved and computational convenience. *α* = *βϕ*_0_/2*f*_0_ is chosen to be 2.0 that corresponds to an experiment value of haptoattractant concentration of fibronectin, which is 200 *μ*g/ml, due to *β*/*f*_0_ = 0.02 ml/*μ*g [29]. A semi-implicit Fourier spectral method is implemented here to have a high spatial resolution to resolve the high-order derivatives in the derived equations as well as the large gradients at the interface region [24]. This method is to treat the linear term implicitly and the nonlinear term explicitly to allow for larger time steps without losing numerical stability and satisfies the requirements for the numerical effectiveness [40]. Furthermore, the Semi-Implicit Backward Differentiation Formula (SBDF) scheme is applied to solve the kinetic equations without a harsh time-step constraint which has the strongest high-modal decay among the second-order multistep methods [41].

To deal with the variable mobility, the right-hand sides of (5), (8), and (9) need to be rewritten as

(10) ∇·M1∇μ1=A1∇2μlr1+sμ1∇·M2∇μ2=A2∇2μlr2+sμ2∇·M3∇m=A3∇2m+sm,

where *μ*_*lr*1_, *μ*_*lr*2_ are linear components of *μ*_1_, *μ*_2_. *s*_*μ*1_ = ∇·(*M*_1_∇*μ*_1_) − *A*_1_∇^2^ *μ*_*lr*1_, *s*_*μ*2_ = ∇·(*M*_2_∇*μ*_2_) − *A*_2_∇^2^ *μ*_*lr*2_, and *s*_*m*_ = ∇·(*M*_3_∇*m*) − *A*_3_∇^2^ *m*. Linear terms *A*_1_∇^2^ *μ*_*lr*1_, *A*_2_∇^2^ *μ*_*lr*2_, and *A*_3_∇^2^ *m* are implicitly treated and nonlinear terms *s*_*μ*1_, *s*_*μ*2_, and *s*_*m*_ are explicitly treated based on the semi-implicit Fourier spectral method. There are various ways to handle the linear component [40, 41]. The numerical stability has been achieved by taking the linear term *μ*_*lr*_ = *c* − *ch* ^2^∇^2^ *c*. Combined with the semi-implicit Fourier spectral method and the SBDF time integration scheme, we obtain the following discretized form:

(11) 32c1n+1−2c1n+12c1n−1=A1Δt2τ1∇2c1n+1+τ1∇2c2n+1−ch112∇4c1n+1−12ch122∇4c2n+1+2P1n−P1n−1,32c2n+1−2c2n+12c2n−1=A2Δtτ2∇2c1n+1+2τ2∇2c2n+1−ch112∇4c1n+1−12ch122∇4c2n+1+2P2n−P2n−1,32mn+1−2mn+12mn−1=A3Δtτ3∇2mn+1+2P3n−P3n−1,32θn+1−2θn+12θn−1=2A4ΔtMdrug∇2θn+1+2P4n−P4n−1,

where *A*_1_, *A*_2_, *A*_3_, and *A*_4_ are the constants and take *A*_1_ = 1, *A*_2_ = 1, *A*_3_ = 1, *A*_4_ = 1, and

(12) P1n=A1Δt∇·M1∇μc1n−ch112∇2c1n−12ch122∇2c2n−αϕ+A1Δt−2τ1∇2c1n−τ1∇2c2n+ch112∇4c1n+12ch122∇4c2n−ηθnc1n,P2n=A2Δt∇·M2∇μc2n−ch222∇2c2n−12ch122∇2c1n+A2Δt−τ2∇2c1n−2τ2∇2c2n+ch222∇4c2n+12ch122∇4c1n−γmnc2n−ηθnc2n,P3n=A3Δt∇·M3∇mn−τ3∇2mn+εc1n−λmn,P4n=A4Δt−Mdrug∇2θn−ηθn.

Taking the Fourier transform to (11), the governing equations of each component are given as

(13) c^1n+1=1DetN42c^1n−12c^1n−1+2P^1n−P^1n−1−N22c^2n−12c^2n−1+2P^2n−P^2n−1,c^2n+1=1Det−N32c^1n−12c^1n−1+2P^1n−P^1n−1+N12c^2n−12c^2n−1+2P^2n−P^2n−1,m^n+1=1Devf4m^n−m^n−1+4P^3n−2P^3n−1,θ^n+1=1Dev4θ^n−θ^n−1+4P^4n−2P^4n−1,

where *k* ^2^ = *k*_1_^2^ + *k*_2_^2^ + *k*_3_^2^, *N*_1_ = 3/2 + 2*A*_1_ *τ*_1_Δ*tk* ^2^ + *A*_1_Δ*tk* ^4^ *ch*_11_^2^, *N*_2_ = *A*_1_ *τ*_1_Δ*tk* ^2^ + (1/2)*A*_1_Δ*tk* ^4^ *ch*_12_^2^, *N*_3_ = *A*_2_ *τ*_2_Δ*tk* ^2^ + (1/2)*A*_2_Δ*tk* ^4^ *ch*_12_^2^, and *N*_4_ = 3/2 + 2*A*_2_ *τ*_2_Δ*tk* ^2^ + *A*_2_Δ*tk* ^4^ *ch*_22_^2^ and Det = *N*_1_ *N*_4_ − *N*_2_ *N*_3_, Dev*f* = 3 + 2Δ*tτ*_3_ *k* ^2^, and Dev = 3 + 4Δ*tM*_drug_ *k* ^2^. The caret “∧” and the subscript *k* stand for the Fourier transform. *c*_1_^*n*+1^, *c*_2_^*n*+1^, *m* ^*n*+1^, and *θ* ^*n*+1^ can be calculated by the followed sequence. First, we compute chemical potentials that correspond to the distributions of *c*_1_^*n*^, *c*_2_^*n*^, *m* ^*n*^, and *θ* ^*n*^. Then we can obtain ${\hat{c}}_{1}^{n+1}$, ${\hat{c}}_{2}^{n+1}$, ${\hat{m}}^{n+1}$, and ${\hat{\theta }}^{n+1}$ from (13). Finally, the new *c*_1_^*n*+1^, *c*_2_^*n*+1^, *m* ^*n*+1^, and *θ* ^*n*+1^ are obtained from ${\hat{c}}_{1}^{n+1}$, ${\hat{c}}_{2}^{n+1}$, ${\hat{m}}^{n+1}$, and ${\hat{\theta }}^{n+1}$ by the inverse Fourier transform. The simulation advances by repeating the procedure.

## 3. Result and Discussion

### 3.1. Simulation Details

**Figure 2:**
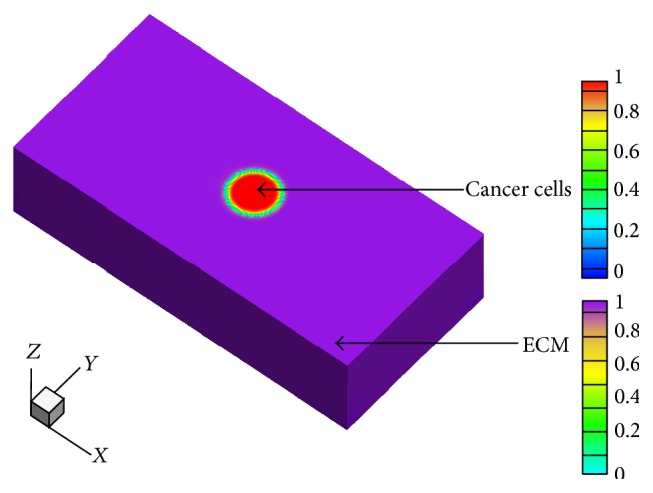
Initial configurations of cancer cells and the ECM.

Here, we quantitatively investigate the characteristics of cancer cell invasion and then systematically analyze the effect of chemotherapy on the invasion process with the developed model. The morphological evolution and migration of cancer cells and the toxicity of chemotherapeutic drug on the normal cell are specifically explored. Initial configurations of cancer cells and the ECM within the domain are illustrated in Figure 2. The domain size is set to be 200 × 100 × 100. The characteristic length is taken to be 3.0 *μ*m and cancer cells are initially positioned at (100,50,50) in the spatial domain. The radius of a cancer cell is considered to be 3.6 × 10^−2^ mm, which gives a typical cancer-cell volume of 1.5 × 10^−5^ mm^3^ per cell [42, 43]. Initial numbers of cancer cells, and normal cells that reside in the ECM, are approximately 5.45 × 10^8^ and 1.30 × 10^11^, respectively. Real time is equal to time scale multiplied by time steps of simulation. Time scale *t*_*c*_ = *L*_*c*_^2^/*M*_0_ *f*_0_ is chosen to be 3.2 s, due to *f*_*o*_ = *h*_*ij*_/*L*_*c*_^2^ = 2.8 that corresponds to an experiment value of the surface energy of poly(dimethylsiloxane) (PDMS), which is 22 ~ 25 mJ/m^2^ [44]. In the following sections, we use the time step instead of real time of simulation to represent the evolution of cancer-cells invasion with chemotherapy.

### 3.2. Diffusion of Chemotherapeutic Drug through Cancer Cells and ECM

**Figure 3:**
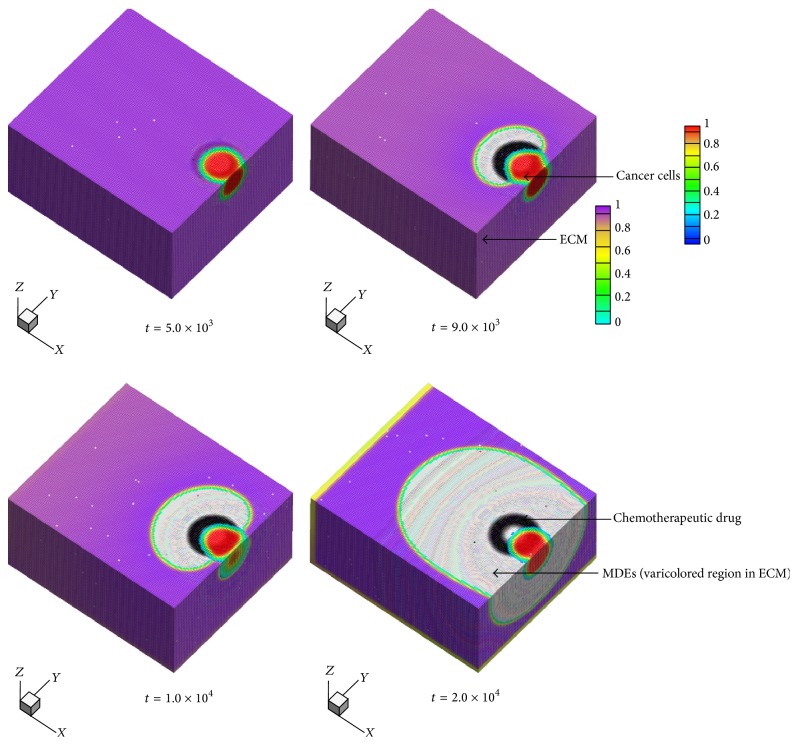
Evolution sequences of cancer-cells invasion with chemotherapy; matrix-degrading enzymes (MDEs) are secreted by cancer cells and chemotherapeutic drug particles surround cancer cells.

We have performed simulations that adequately expound the diffusion process of chemotherapeutic drug through both of cancer cells and the ECM before investigating the efficacy including the systemic effect of the drug.  Figure 3 shows cross-sectional views of cancer-cells invasion with chemotherapeutic drug in the computational model. The color bar presents that the field variables of cancer cells and the ECM are changing from 1.0 to 0.0. One has that *c*_1_ = 1.0 (red color); *c*_1_ = 0.0 (blue color); *c*_2_ = 1.0 (purple color); *c*_2_ = 0.0 (green color), applying *c*_1_ = 1.0 with *c*_2_ = 0.0 and *c*_1_ = 0.0 with *c*_2_ = 1.0. The red area at the center of the domain represents cancer cells and the purple color in the surrounding area depicts the normal matrix. In the process of cancer-cells invasion, MDEs play an important role in degrading the ECM. Cancer cells secrete MDEs that destroy the normal tissue. As shown in Figure 3, MDEs are secreted by cancer cells and expressed as the varicolored region in the ECM. The number of MDEs is increasing as time increases because cancer cells secrete MDEs continuously. Chemotherapeutic drug that is described by a doughnut-shape with a dark gray color surrounds cancer cells. The mathematical model allows the drug to diffuse through both cancer cells and the ECM. Chemotherapy aims to kill abnormal or cancer cells but the normal matrix is also destroyed by the drug, which is plausibly simulated with the developed model. The clear observations supply a particularly intuitive approach and an easy way for understanding the detailed process of invasion with chemotherapy. Specialized protrusions, especially, include chemotherapeutic drug and MDEs secreted by cancer cells which are both incorporated in the dynamic model for comprehensive analysis. The three-dimensional model provides insights into understanding the mechanisms of each component in cancer-cells invasion viewing microstructural evolution and focuses our attention on specialized protrusions involved in multicomponent of cancer-cells invasion and chemotherapy treatment. Nevertheless, one- and two-dimensional models still provide important insights into cancer-cells invasion which limits our understanding of multimechanisms and different migration strategies in cancer-cells invasion. It has been possible to overcome these difficulties by creating the three-dimensional model, which presents the real state of cancer-cells invasion more precisely.

**Figure 4:**
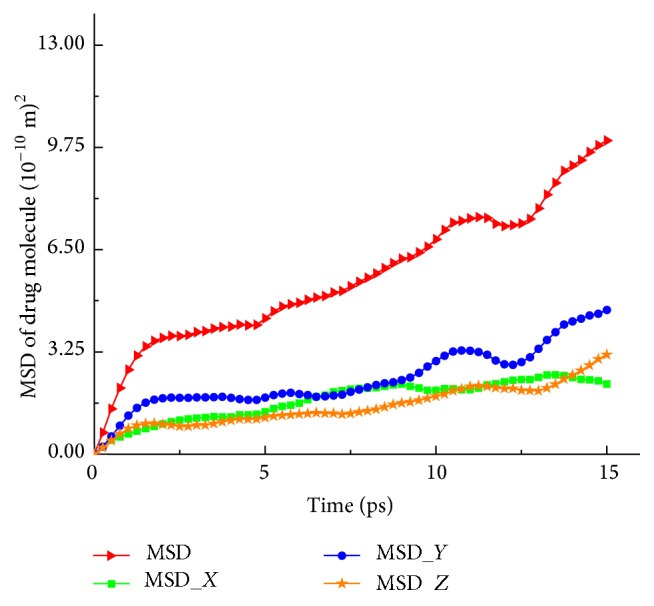
Mean-squared displacement (MSD) obtained from the trajectory of the drug molecules in MD simulation.

**Figure 5:**
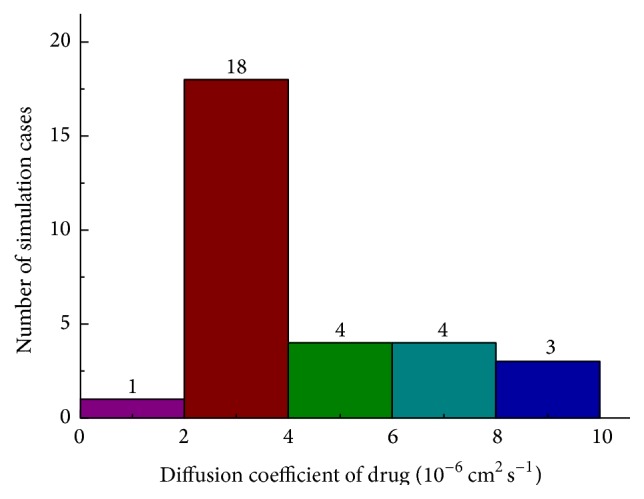
Number of cases in different ranges of diffusion coefficient.

MD simulations have been carried out to study the diffusion process of chemotherapeutic drug. The diffusion coefficient can be calculated from the trajectory of the drug. MSD has been plotted as a function of time in Figure 4. The diffusion coefficient of the drug is proportional to the slope of MSD. Thirty cases have been simulated to get the accurate average value of diffusion coefficients. As shown in Figure 5, the diffusion coefficient in 60% cases is approximately 2.0 × 10^−6^ ~ 4.0 × 10^−6^ cm^2^ s^−1^. The average value of diffusion coefficient of the drug, *M*_drug_, is 4.13 × 10^−6^ cm^2^ s^−1^. The calculated value is consistent with previous study that presented the base value of diffusion coefficient of diffusant in the cellular biological medium approximately 2.0 × 10^−6^ cm^2^ s^−1^ [45]. They proposed an expression for the local effective diffusion coefficient as *D*_*Aβ*_ = *λ*_*β*_ *D*_*Aυ*_; here *D*_*Aυ*_ represents diffusion coefficient in water and the base value of it is 20.0 × 10^−6^ cm^2^ s^−1^. *λ*_*β*_ is (0.01–0.99)^*h*^ that is a function of the composition and fundamental geometric and physicochemical system properties, including the size of solute molecules, the size of extracellular polymer fibers, and the mass permeability of cell membrane; the base value of *λ*_*β*_ is mentioned and equals 0.1. They made their effort on representing the local effective diffusion coefficient of diffusant in the cellular biological medium by using the generalized function that could not provide the accurate value of diffusion coefficient of a particular diffusant in a specified medium. The other related work indicated that the diffusion coefficient of theophylline in the polymeric membrane is affected by concentration of the polymer and temperature [46]. The final diffusion coefficient of this material was 5.00 × 10^−6^ cm^2^ s^−1^ at 25°C, but this value was also obtained from the numerical model. And detailed analysis of diffuse process was depending on unique structure of materials and system. Based on the above researches, it is necessary to perform detailed atomic simulation to get diffusion coefficient of the particular anticancer drug in the specified medium as accurate as possible. Our atomic simulation is primary depending on considering the detailed structure of systems and run at room temperature with constant volume and a fixed density of the polymer molecules system. We believe that the developed computational model can provide a reliable and efficient method to study the chemotherapy.

**Figure 6:**
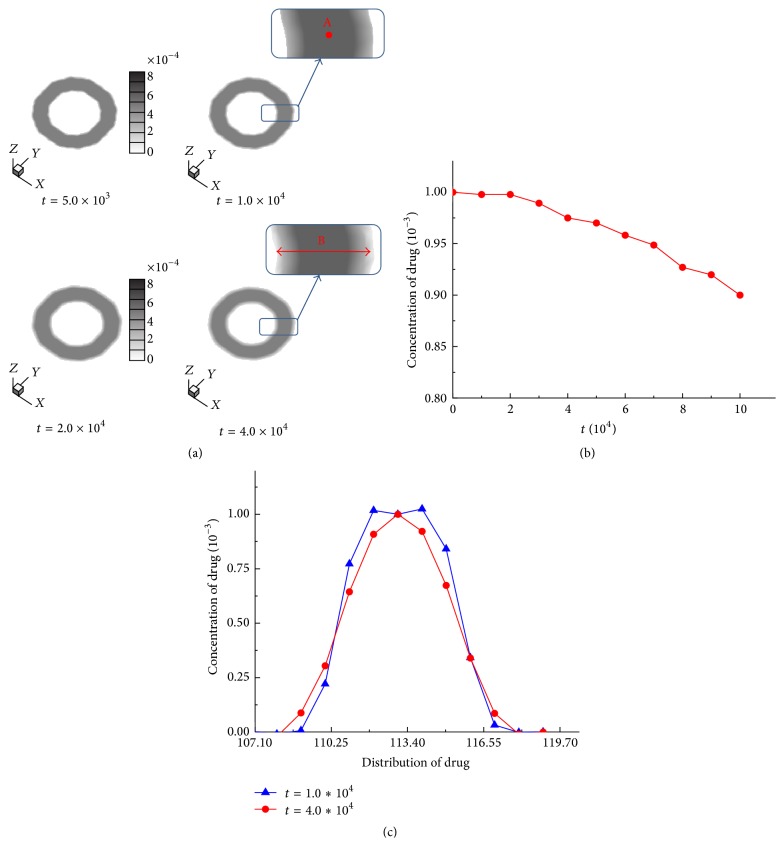
(a) Diffusion of chemotherapeutic drug, (b) evolved concentration of chemotherapeutic drug in the treatment, and (c) distribution of chemotherapeutic drug in the diffusion process.

Taking the calculated diffusion coefficient of chemotherapeutic drug with MD simulation into the developed three-dimensional model of cancer-cells invasion, the diffusion process of chemotherapeutic drug is simulated as shown in Figure 6(a). The concentration of chemotherapeutic drug decreases during the chemotherapy treatment. The concentration data of the drug which is obtained from the middle region of the doughnut-shape and marked in point “*A*” is shown in Figure 6(b) and it demonstrates that the highest concentration of the drug occurs at *t* = 0.0 and the concentration is decreasing as the time increases due to natural decay phenomenon of chemotherapeutic drug. A comparison of the distribution of chemotherapeutic drug in the region “*B*” at *t* = 1.0 × 10^4^ and *t* = 4.0 × 10^4^ is presented in Figure 6(c). It is observed that the concentration of the drug at the center region decreases and has a wider distribution as time increases since the drug diffuses into cancer cells and normal tissues. The presented simulation results adequately explain the dynamic characteristics of chemotherapeutic drug, which incorporate the atomic-scale insight obtained from atomic simulations.

### 3.3. Effect of Chemotherapeutic Drug on Invasion of Cancer Cells

**Figure 7:**
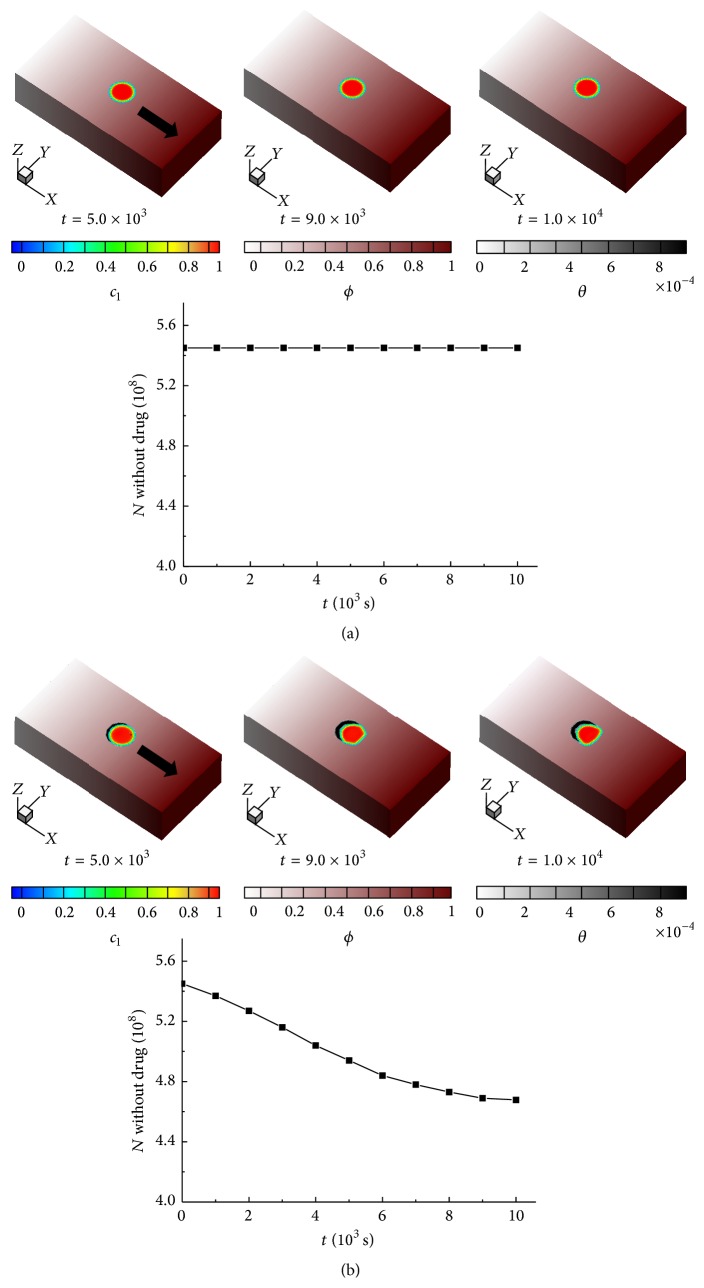
Morphological and the number changes of cancer cells (a) without chemotherapeutic drug and (b) with chemotherapeutic drug.

To quantify the effect of chemotherapeutic drug on cancer cells, we investigate the morphological changes of cancer cells without and with chemotherapeutic treatment which are shown in Figures 7(a) and 7(b), respectively. As shown in Figure 7(a), the color change from red to blue means that the variable of cancer cells is changing from 1.0 to 0.0. The change of dark red color to white color represents haptoattractant density gradient in the ECM. Higher haptoattractant density gradient in the dark red color is assigned on the right region of the ECM to induce cancer cells moving from the left to the right side. In Figure 7(b), chemotherapeutic drug is used in the system; the color change from gray to white indicates the different concentration of chemotherapeutic drug which is initially assigned as a doughnut-shape surrounding cancer cells.

As shown in Figures 7(a) and 7(b), the morphological shape of cancer cells and the number of cancer cells are changed as the chemotherapeutic drug is introduced. The population of cancer cells with chemotherapy changes its shape from spherical to deformed along the distribution of chemotherapeutic drug that hinders cancer-cells migration. As shown by two graphs, the total number of cancer cells with chemotherapeutic drug is decreased by about 14.16%. *N* denotes the number of cancer cells. The measured velocity of cancer-cells migration is 3.22 *μ*m/hr but with the presence of chemotherapeutic drug, the velocity decreases to 2.83 *μ*m/hr when the time steps of simulation are 1.0 × 10^4^. The effects of the ECM on the invasive behavior of cancer cells have been introduced by an experimental work [47]. Experimentally observed average velocity of human pancreatic cancer cells (HPAF-II) in the ECM is 5.5 ± 1.7 *μ*m/hr. The agreement is reasonably convincing.

### 3.4. Capacity of Chemotherapy to Destroy Cells

**Figure 8:**
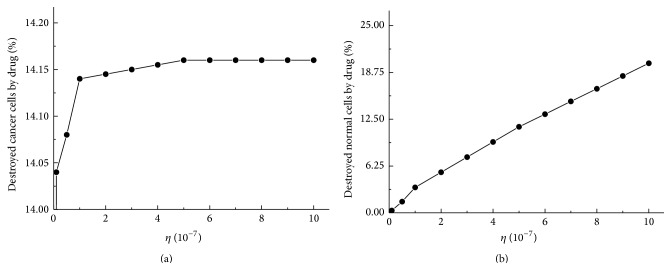
Reduction ratio of (a) cancer cells and (b) normal cells with various capacity of chemotherapeutic drug on destroying cells, *η*, at *t* = 2.0 × 10^4^.

Chemotherapy plays a role in not only affecting the morphology of the population of cancer cells but also killing cancer cells. It is also necessary to consider the effect of chemotherapy on the normal cells due to the toxicity of the drug. Many researches have made the efforts to study chemotherapy treatment in tumor cells [2, 48]. The work of delivery of chemotherapeutic agents in cancer introduced by the mathematical model revealed that approximately 60% and 80% of the initial tumor size and normal cell population reduced when the chemotherapy is performed in the treatment [2]. The seriously destroyed phenomenon on normal cells requires designing a reliable method to kill maximum number of cancer cells and minimum number of normal cells. Thus, we study the influence of chemotherapeutic drug to suggest the enhanced chemotherapy that minimizes cancer cells while limiting the toxicity on normal cells. The capacity of chemotherapeutic drug on destroying cells is represented by a parameter *η*. The initial number of cancer cells and normal cells in the domain is considered to be 5.45 × 10^8^ and 1.30 × 10^11^, respectively. Cancer cells killed by chemotherapeutic drug from *η* = 0.0 to *η* = 10.0 × 10^−7^ at *t* = 2.0 × 10^4^ are presented in Figure 8(a). As increasing the parameter *η*, more cancer cells are destroyed by a potent drug. It is observed that at most 14.16% of cancer cells are killed by the drug when the parameter *η* is greater than or equal to 5.0 × 10^−7^. Even though the parameter is larger than 5.0 × 10^−7^, the number of cancer cells killed by the drug does not considerably increase. Simulation results suggest the minimum efficacy of the drug with a particular amount in the cancer-cells invasion. In Figure 8(b), the reductions of normal cells with various values of parameter *η* are presented. Approximately 11.48% of normal cells are destroyed by the drug with *η* = 5.0 × 10^−7^ and the reduction of normal cells increases to 19.97% with *η* = 10.0 × 10^−7^. From the observation with Figure 8(a), the number of cancer cells is not appreciably altered with the parameter from *η* = 5.0 × 10^−7^ to *η* = 10.0 × 10^−7^. The performed simulations demonstrate that the appropriate value for the capacity of chemotherapeutic drug is about in the range of 1.0 × 10^−7^ ~ 5.0 × 10^−7^ to ensure that the maximum number of cancer cells and the minimum number of normal cells are destroyed under chemotherapy treatment. We consider a specific drug that corresponds to a certain diffusion coefficient and parameter *η*. The parameter *η* can be manipulated by a proper carrier by the development of nanotechnology [49]. Nanoparticles as a carrier provide a new mode of delivery of anticancer drug to enhance the capacity on destroying cancer cells. The motivation of the development of neoadjuvant chemotherapy is to supply an efficient and reliable drug with applicable value of the parameter *η* on killing cells. If we consider the totally different kinds of drug that have obviously different mobility from other drugs, by recalculating the diffusion coefficient with the presented process, the expeditious analysis of chemotherapy process can be carried out with the developed model. For drugs with the recognized mobility, it would be prompt investigation. As presented by simulation results, the ECM is destroyed more seriously than cancer cells since the ECM were killed by chemotherapeutic drug and MDEs secreted by cancer cells. In addition, it is experimentally observed that drug molecules diffuse with the higher diffusivity in the ECM than in cancer cells [7, 8], because cancer cells are densely packed in islet, which leads the drug particles hard to delivery to the inside. Thus, the destroyed phenomenon on the ECM is more obvious than cancer cells, which is clearly demonstrated, and a reliable parametric study provided the developed model for the capacity of chemotherapeutic drug on killing cells.

### 3.5. Effect of Chemotherapeutic Drug on Cancer-Cells Migration

**Figure 9:**
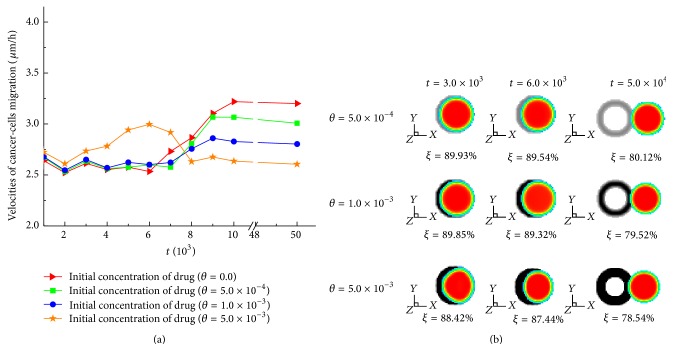
(a) Velocities of cancer-cells migration with various concentrations of chemotherapeutic drug. The initial concentrations of drug are 0.0, 5.0 × 10^−4^, 1.0 × 10^−3^, and 5.0 × 10^−3^, respectively. (b) Position of cancer cells with different initial concentrations of drug. *ξ* = (Current volume of cancer cells/Initial volume of cancer cells) × 100%.

The velocity of cancer cells as well as the destroyed amount of cancer cells could be critical for the design of chemotherapy especially when the location of cancer cells is fatal. Simulations with various initial concentrations of chemotherapeutic drug are performed to provide analytical information of the capability of the drug in cancer treatment. The velocities of cancer cells with various concentrations of chemotherapeutic drug are shown in Figure 9(a) and those at specific time steps are listed in Table 2. The maximum velocity of cancer cells is obtained with high or low concentration of drug depending on the simulation time. Before about *t* = 6.0 × 10^3^, the maximum velocity of cancer cells is observed with the high initial concentration of drug since more ECM is degraded and more space for cancer cells migration is generated. Cancer cells, however, are degraded seriously with high initial concentration of drug. The position of cancer cells with different initial concentrations of drug is demonstrated in Figure 9(b). *ξ* represents the ratio of current volume of cancer cells to the initial volume of the population of cancer cells. Within the region with the drug, cancer cells with the higher concentration of drug are more destroyed and quickly migrate through the more seriously destroyed ECM region. After *t* = 6.0 × 10^3^ when cancer cells start to escape from the region with the drug, the velocity of cancer cells with the low concentration of drug increases substantially while that with high concentration of drug decreases. Some experimental observations revealed that cancer cells could migrate through the connective tissues by their own mobility and response to the chemoattractant gradient assigned in the tissue matrix [50, 51]. The directed migration could determine that cancer cells migrate and escape from the region with the toxic effect of commonly used cancer drugs. Thus, we make the effort to incorporate this effect and investigate the velocities of cancer cells with various concentrations of chemotherapeutic drug before and after escaping from the region with the drug. Cancer cells with the high concentration of drug are degraded more seriously and secrete less MDEs than those with the low concentration of drug. Thus, after escaping from the region with the drug, cancer cells with the high concentration of drug have difficulty in generating space for invasion by degrading the ECM. Hence, the region where the drug is assigned as well as the amount of it is essential for the high efficacy of chemotherapy.

## 4. Conclusion

In this paper, we have presented a multiphysics and multiscale model of chemotherapy and studied the invasion process of cancer cells. Cancer cells secreting MDEs, degrading ECM, and chemotherapeutic drug are systematically incorporated and the multiple mechanisms effectively integrated. Atomic simulations have been carried out and enhanced the reliability of the simulated diffusion process of chemotherapeutic drug and corresponding estimation of the efficacy of drug treatment. Simulation results demonstrate practical morphological evolution and invasive kinetics of cancer cells under chemotherapy treatment, which is limited with one- or two-dimensional models. Moreover, the simulation results performed with the developed model give insights that chemotherapeutic drug could change the morphological shape of cancer cells to prevent migration and decrease the number of cancer cells. Furthermore, the quantitative analysis provides the existence of an optimal amount of chemotherapeutic drug on improving chemotherapy treatment.

## Figures and Tables

**Table 1:** Parameters used in the system.

Parameter	Definition	Value
*γ*	Degradation rate of ECM by MDEs	1.0 × 10^−6^
*ε*	Released rate of MDEs by cancer cells	1.0 × 10^−6^
*λ*	Natural degradation rate of enzymes	1.0 × 10^−6^
*α*	Significance of the haptotaxis	2.0
*ch*_*ij*_	Cahn number	1.0

**Table 2:** Velocities of cancer-cells migration at different time steps (*μ*m/hr).

Concentration	0	2000	4000	6000	8000	10000	50000
0.0	0	2.52	2.55	2.53	2.87	3.22	3.20
5.0 × 10^−4^	0	2.53	2.56	2.59	2.81	3.07	3.01
1.0 × 10^−3^	0	2.55	2.57	2.60	2.76	2.83	2.80
5.0 × 10^−3^	0	2.61	2.78	3.00	2.63	2.64	2.60
